# Retrospective analysis of 85 cases of intermediate-risk gastrointestinal stromal tumor

**DOI:** 10.18632/oncotarget.14359

**Published:** 2016-12-29

**Authors:** Yang Fu, He Hao, Luwei Guo, Ge Yang, Xiefu Zhang

**Affiliations:** ^1^ Department of General Surgery, The First Affiliated Hospital of Zhengzhou University, Zhengzhou 450052, China; ^2^ Department of Ophthalmology, The First Affiliated Hospital of Zhengzhou University, Zhengzhou 450052, China

**Keywords:** GIST, intermediate-risk, imatinib, retrospective analysis, RFS

## Abstract

**Background & Aims:**

A significant benefit of imatinib adjuvant therapy for patients with high risk gastrointestinal stromal tumors (GIST) has been confirmed. However, the effect of imatinib adjuvant therapy for intermediate-risk GIST has not been well studied. In this article, we compare differences of recurrence-free survival (RFS) rates between patients with intermediate-risk GIST who accepted imatinib adjuvant therapy and those who did not.

**Method:**

A retrospective study of intermediate-risk GIST was conducted in the First Affiliated Hospital of Zhengzhou University, China. The pathology reports of 112 patients who had been treated by surgery showed intermediate-risk GIST. The treatment and control groups were designed according to the administration of imatinib adjuvant therapy (≥1 year). Survival and recurrence data were collected and RFS of each group was calculated.

**Results:**

Eighty fivepatients with intermediate-risk GIST were followed up. Thirty of them (treatment group) accepted imatinib adjuvant therapy over 1 year. Through comparing the RFS of the two groups, we established that there was no statistically significant difference in RFS rates (P=0.940).

**Conclusion:**

There is no significant benefit for patients with intermediate-risk GIST to accept imatinib adjuvant treatment.

## INTRODUCTION

Gastrointestinal stromal tumors (GIST) are the most common sarcoma of the intestinal tract [1–5], which are believed to originate from the interstitial cells of Cajal, the pacemaker cells of the gastrointestinal tract [6, 7]. They represent approximately 1% to 2% of all the alimentary malignancies [8]. Gastrointestinal stromal tumors are usually found in the stomach or the small intestine but can occur at any site along the gastrointestinal tract and rarely elsewhere within the abdominal cavity [5, 6, 9]. The mainstay of treatment for localized, primary GIST has been surgical resection [10–14]. However, the results of surgery alone have been inadequate, with up to 50% of patients developing tumor recurrence within 5 years after the surgery and eventually dying of disease [9, 15]. Unparalleled clinical efficacy recently demonstrated in prolonging recurrence-free survival (RFS) for patients with higher risk has brought it to the center stage [1, 2]. Meanwhile, both of the National Comprehensive Cancer Network (NCCN) and the European Society for Medical Oncology (ESMO) guidelines recommend using adjuvant imatinib for at least 3 years in patients with a significant risk (NCCN) or high risk (ESMO) of recurrence, and not to consider adjuvant imatinib when the risk of recurrence is low. However, for patients with intermediate-risk of recurrence the NCCN guidelines do not give any recommendation. ESMO guidelines suggest room for shared decision making between patients and physicians, but without further-detailed approaches [13, 14].

Although Chinese Expert Consensus recommends adjuvant imatinib for 1 year when the risk of recurrence is considered intermediate, no studies have been done to determine if it is the best therapeutic schedule. Therefore, in this paper we will discuss the curative effect of imatinib postoperative adjuvant treatment on intermediate-risk GIST patients.

## RESULTS

### Recurrence and metastases

In the control group, there were 3 patients who had metastatic liver after discontinuing the treatment. Two of them received higher doses of imatinib therapy only (800mg/day) for the multiple metastases, and the other patient received treatment of radiofrequency ablation for solitary metastasis.

In the treatment group, 2 patient experienced recurrence or metastasis. One of them had a local, recurrent metastasis in the abdomen who received surgical treatment and imatinib therapy (400mg/day) after surgery. The other patient had hepatic metastasis and did not receive any treatment on account of economic factors.

### Survival analysis and statistics

**Figure 1:**
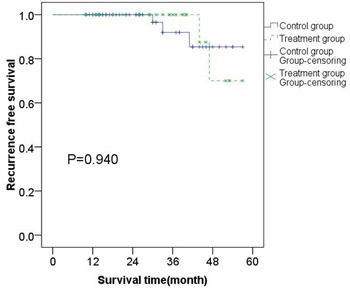
Kaplan–Meier estimates of the RFS of 85 patients with intermediate-risk GISTs There was no statistical difference of RFS between treatment group and control group.

The follow-up period ended on July 1, 2016, and no deaths in the 85 patients were reported. The RFS in the treatment group in the 1st, 2nd, 3rd and 4th year was 100%, 100%, 100%, and 81% respectively and that in the control group in the 1st, 2nd, 3rd, and 4th year was 100%, 100%, 93%, 85% respectively (Table 1). Through comparing the RFS in both groups we established that there was no statistically significant difference in RFS between two groups (Figure 1 P=0.940). This finding, apparently, indicates that there is no significant benefit for patients with intermediate-risk GIST to accept imatinib adjuvant treatment.

**Table 1:** RFS in the 1st, 2nd, 3rd, and 4th year of treatment group and control group respectively

	1^st^ year RFS	2^nd^ year RFS	3^rd^ year RFS	4^th^ year RFS
Treatment group	100%	100%	100%	81%
Control group	100%	100%	93%	85%
All patients	100%	100%	96%	83%

### Other influencing factors analysis

**Figure 2:**
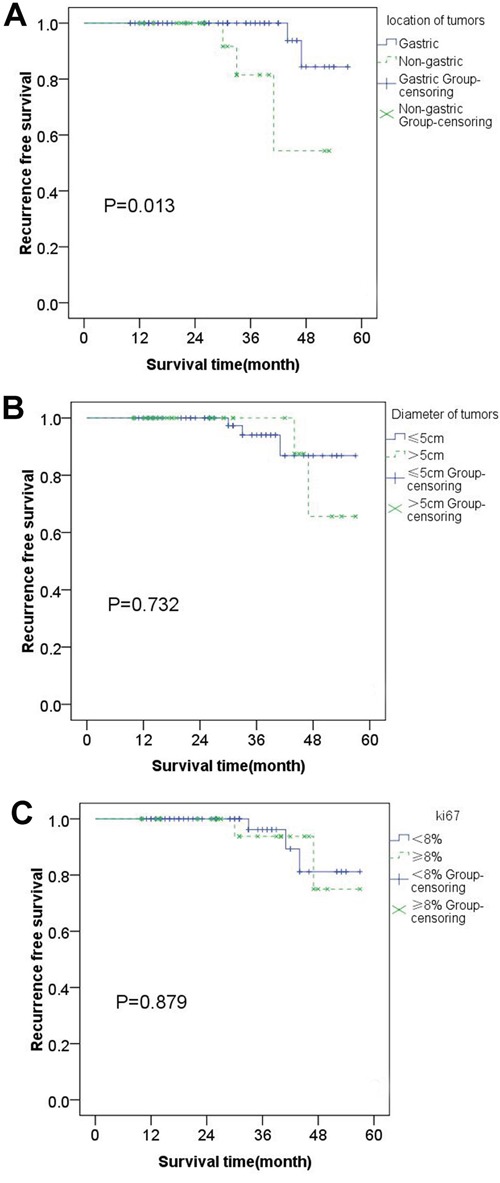
Kaplan–Meier estimates of the RFS of 85 patients: RFS was significantly lower in patients with tumors located in stomach than that located in other site A There is no statistical difference between two groups which were grouped based on tumor size and Ki-67 respectively B & C.

The most important single factor for recurrence is high tumor mitotic count and non-gastric site of origin [16]. Simultaneously, increasing evidence indicates that large tumor size and tumor rupture are also independently associated with RFS [15, 17–19]. However, when is referred to the recurrence risk for intermediate-risk GIST patients, whether the origin site, size, as well as mitotic figures, can also be independent factors? Hence, we respectively analyzed each individual factor impact on the crisis in the prognosis of GIST in the 85 patients, who were divided into groups according to the site (gastric or non-gastric), size (≤5cm or >5cm) and mitotic figure (≤5/50HPFs or >5/50HPFs). First, all patients were settled in two groups according to the primary site of the tumor (gastric or non-gastric). Among them, the primary site of the tumor in 62 patients (72.94%) was located in the stomach, and in 23 patients (27.06%) was not. In these two groups, the number of patients with metastasis were 2 (gastric) and 3 (non-gastric) respectively. There is a statistical difference between these two groups (Figure 2A p=0.013) and it means primary site of tumor is an independent factor affecting prognosis of intermediate-risk GIST. According to the NIH standards, for the intermediate-risk GIST, regardless of the site of the tumor, if the diameter is longer than 5cm, the mitotic figure is less than 5/50HPFs and vice versa. So grouping by either of these two factors has the same result. We chose tumor size as a factor of grouping for statistical analysis and we could see that there was no statistical difference (Figure 2B P=0.732). Therefore, we could draw a conclusion that the tumor size and mitotic figures are not independent factors affecting the prognosis of intermediate-risk GIST.

In addition, mutation site Ki-67, genome index (GI) can also be independently correlated with the prognosis of GIST [14, 20, 21]. Considering that the data of gene type and gene index of tumors were limited by the results of this examination, we compared the Ki-67 value of the inter-media risk GIST alone. In this categorized scheme, 8% was chosen as a boundary on account that Ki-67>8% was deemed to relate to poor prognosis in GIST [20]. There were 58 patients with Ki-67≤8% and 27 patients with Ki-67>8% (Figure 2C P=0.879) demonstrating that Ki-67 cannot be an independent factor in the inter-media risk GIST.

## DISCUSSION

In recent years, the incidences of GIST have been on the rise [22–24]. The comprehensive treatment based on surgery has become a routine treatment method [11, 25–29]. As a means of adjuvant therapy, imatinib has been proven effective for the high-risk patients in a number of studies [12]. The clinical trial of Z9001 suggested that RFS of high-risk patients who received 1 year of adjuvant therapy was significantly higher than that of the patients in the placebo group [1, 30]. Moreover, in the SSGXV III experiment for high-risk GIST patients, OS and RFS to 3 years of adjuvant therapy gained more significant benefits than 1 year of adjuvant treatment [2, 31]. Thus, for high-risk GIST patients, it can be concluded that longer adjuvant therapy may be associated with a better prognosis. However, for intermediate-risk patients whether adjuvant therapy with imatinib will have a better outcome was not clear. The EORCT 62024 study indicated that there was no significant difference in RFS between the patients receiving imatinib adjuvant therapy for two years and patients in the observational arm [32], which is very similar to our conclusion.

In our study, we retrospectively analyzed patients with intermediate-risk GISTs. We came to a conclusion that patients with intermediate-risk GISTs didn't gain significant benefit from imatinib adjuvant therapy. Also, analyzing factors that may have influenced RFS of intermediate-risk GISTs we found that only the locations of tumor sites were independently associated with GIST.

According to the online information from National Bureau of Statistics of the People's Republic of China, the per capita disposable income in China was CNY 21,966 (about USD 3,285.1) in 2015. The annual cost of 400 mg daily imatinib therapy was CNY 72,000 (about USD 10,767.9) in 2015. Besides, the costs of Glivec are not covered by medical insurance schemes in China. This means it takes three people's income to approximately afford imatinib therapy for one patient. This is the main reason why the number of the participants in the control group was larger than the number of participants in the treatment group.

Analysis shows that there was no difference in the RFS between both groups and it was 100% in the first two years. In the third year, the RFS of the control group began to decline while that in the treatment group did not; whereas the RFS in the treatment group was lower than that in the control group in the fourth year. Further analyzing the results it can be seen that by the end of the fourth year the number of the patients with recurrent symptoms was 2 in the treatment group, compared with 3 patients in the control group. Nevertheless, the total number of patients in the treatment group was less than the number of the patients in the control group, which in return resulted in lower RFS in the treatment group.

Analyzing the results of the recurrence timing, the main reason for all patients not to experience recurrence or metastasis in the first 2 years after surgery might have been on account of having intermediate-risk GISTs and accepting standard R0 resection. This may have contributed to a better prognosis. The first patient with recurrence in the control group was in the 30th month of the operation, while the first patient in the treatment group with an adjuvant therapy of 12 months was in the 44th month. Coincidentally, the difference between the two groups first patients’ recurrence time was 14 months. It can be speculated that, although the RFS of the two groups had no significant difference, the treatment group relapsed later than the control group. More work should be done to prove whether patients with adjuvant therapy can prolong the time of the first relapse.

Considering the fact that RFS was not significantly different in the way of dividing patients by imatinib adjuvant therapy, we also stratified them based on the tumor site (gastric or non-gastric), tumor size (≤5cm or>5cm) and Ki-67 (≤8%or>8%) respectively. Regrettably, we found that there was a statistical difference in one pair (tumor site) and no statistical significance in two pairs (tumor size & Ki-67). It suggested that the figure based on the tumor site (gastric or non-gastric) pointed to differences (Figure 2A P=0.013) which showed that the primary site of the tumor was an independent factor associated with the prognosis of intermediate-risk GISTs. However, the tumor size or Ki-67 may not be an independent prognostic factor in the patients with intermediate-risk GIST (Figure 2B P=0.732; Figure 2C P=0.879).

After an initial response to imatinib, the vast majority of patients were eventually lead to secondary resistance. For the intermediate-risk GIST patients, since they cannot benefit from adjuvant therapy, it is worth thinking whether it is necessary to treat them with adjuvant therapy. If secondary resistance occurs, it will increase the difficulty of treatment. Even the EORCT 62024 study reported that adjuvant imatinib for intermediate-risk GIST cannot affect IFFS (imatinib failure–free survival; i.e., time to resistance to imatinib) [32], which means it may not increase the risk of secondary resistance. Their classification, though, is according to 2002 National Institutes of Health, which is different from the classification we used.

To sum up, imatinib adjuvant therapy in patients with intermediate-risk GIST cannot achieve significant gains in 3 years. Moreover, factors such as the first recurrence time and secondary drug resistance, suitability of adjuvant therapy and appropriate treatment time are worth considering. What we want most is to maximize the benefits of the patient. It is meaningful to identify other influential “high-risk factors” (e.g. genotype) of intermediate-risk GIST and explore further treatment options.

## PATIENTS AND METHODS

### Classification standard

The mitosis count, tumor size, tumor site and tumor rupture are important prognostic predictors in GIST [9, 33]. Although both NCCN and ESMO guidelines classify GIST according to these four criteria, there is no consensus on which risk classification system is best suited for determining patients’ risk of recurrence [34]. Modified NIH criteria based on NIH consensus criteria is wildly accepted as risk-stratification scheme for GIST and four categories from very low to high risk are used to predict prognosis of GIST patients [20, 35]. Since NIH grading standard is widely used, we chose it in our study.

### Patients

This study was approved by the First Affiliated Hospital of Zhengzhou University. All procedures performed in studies involving human participants were in accordance with the 1964 Declaration of Helsinki and its later amendments or comparable ethical standards. Informed consent was waived by the committee because of the retrospective nature of the study.

**Figure 3:**
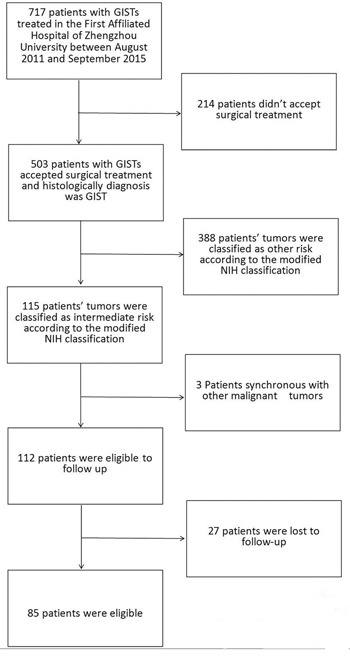
The process of screening medical records of patients

The medical records of all patients with GISTs were collected in the First Affiliated Hospital of Zhengzhou University between August 2011 and September 2015. The inclusion criteria were as follow: 1. patients had a histological diagnosis with primary GISTs and had undergone surgical treatment; 2. the tumors were classified as intermediate-risk according to the modified NIH classification; 3. patients with other malignancies and insufficient medical charts were excluded. A total of 503 patients had had a complete surgical resection, and pathological diagnosis after the operation was GIST. Among them, 115 patients had intermediate-risk GIST, whereas 3 patients with other coexisting malignant tumors were excluded and 27 patients failed in follow-up. Ultimately, 85 patients were eligible for the test (Figure 3).

### Statistical method

Data were processed using SPSS 17.0 for Windows. The primary objective was RFS, defined as the time from patient registration to the development of tumor recurrence or death due to any cause. Survivals between groups were compared using the Kaplan-Meier life-table method and unstratified log-rank test (P-values). The P values were considered to be statistically significant at the level of 5%

### Patient information

Data of 85 eligible patients with intermediate-risk GIST were collected. Their median age was 60.5 years (range 28-77 years). The detailed baseline characteristics are summarized in Table 2. Besides, 3 patients had a postoperative complication, one of which had gastric volvulus and the other two had an intestinal obstruction. All of them received surgical treatment and recovered.

**Table 2:** Clinicopathologic Characteristics of 85 Patients of intermediate-risk Gastrointestinal Stromal Tumors

	Number	Percentage	Treatment group	Control group
**Gender**				
Male	33	38.80%	13	20
Female	52	61.20%	17	35
**Age, y**				
≤50	12	14.10%	4	8
>50	73	85.90%	26	47
**Tumor site**				
Stomach	62	72.90%	23	39
Small intestine	20	23.50%	6	14
Large intestine	3	3.60%	1	2
**Tumor size, cm**				
≤5	41	48.20%	18	23
>5	44	51.80%	12	32
**Mitotic figure, /50HPFs**				
≤5	44	51.80%	12	32
6-10	41	48.20%	18	23
**Ki-67**				
≤8%	56	65.90%	22	34
>8%	29	34.10%	8	21
**Mutation status (Σ=17)**				
KIT exon 9	1	5.88%	0	1
KIT exon 11	12	70.59%	4	8
PDGFRA exon18	1	5.88%	0	1
Wild type	3	15.65%	0	3
**IHC (positive)**				
DOG-1(Σ=79)	76	96.20%	27	49
CD117(Σ=83)	83	100%	30	55
CD34(Σ=82)	73	89.02%	29	44

**Abbreviations:** IHC=Immunohistochemistry HPFs=High power field

### Patient groups

**Figure 4:**
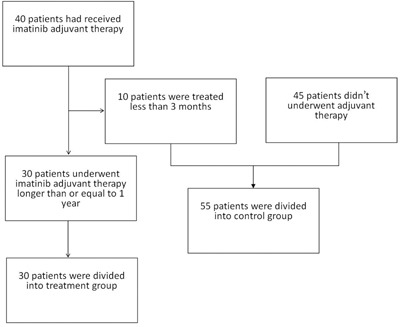
The process of grouping for patients

Two groups (treatment group and control group) were designed for comparison. The treatment group was defined as patients who had undergone imatinib adjuvant treatment longer than or equal to 1 year, while the control group included patients who had undergone imatinib adjuvant treatment shorter than 3 months or never. Forty-five patients had never received adjuvant therapy, so they were included in the control group. The other 40 patients had received imatinib adjuvant therapy but 10 of them who were treated less than 3 months because of side effects or economic factors were included in the control group. Finally, the treatment group was limited to 30 patients (Figure 4). Among them, 1 patient had been treated for 10 months by that time and the treatment was still underway; 5 patients had been treated for one year; 24 patients had been treated for more than one year (ranging from 23 months to 57 months), and the median duration of treatment was 34 months.
